# Magnetic levitational bioassembly of 3D tissue construct in space

**DOI:** 10.1126/sciadv.aba4174

**Published:** 2020-07-15

**Authors:** Vladislav A. Parfenov, Yusef D. Khesuani, Stanislav V. Petrov, Pavel A. Karalkin, Elizaveta V. Koudan, Elizaveta K. Nezhurina, Frederico DAS Pereira, Alisa A. Krokhmal, Anna A. Gryadunova, Elena A. Bulanova, Igor V. Vakhrushev, Igor I. Babichenko, Vladimir Kasyanov, Oleg F. Petrov, Mikhail M. Vasiliev, Kenn Brakke, Sergei I. Belousov, Timofei E. Grigoriev, Egor O. Osidak, Ekaterina I. Rossiyskaya, Ludmila B. Buravkova, Oleg D. Kononenko, Utkan Demirci, Vladimir A. Mironov

**Affiliations:** 1 Laboratory for Biotechnological Research “3D Bioprinting Solutions”, Moscow, Russia.; 2 A.A. Baikov Institute of Metallurgy and Material Science, Russian Academy of Sciences, Moscow, Russia.; 3 P.A. Hertsen Moscow Oncology Research Center, National Medical Research Radiological Center, Moscow, Russia.; 4 Peoples' Friendship University of Russia (RUDN University), Moscow, Russia.; 5 Riga Technical University, Riga, Latvia.; 6 Joint Institute for High Temperatures, Russian Academy of Sciences, Moscow, Russia.; 7 Susquehanna University, Selinsgrove, PA, USA.; 8 National Research Center “Kurchatov Institute”, Moscow, Russia.; 9 Imtek Ltd., Moscow, Russia.; 10 Central Research Institute for Machine Building, Korolev, Moscow Region, Russia.; 11 Institute of Biomedical Problems, Russian Academy of Sciences, Moscow, Russia.; 12 Yu.A. Gagarin Research & Test Cosmonaut Training Center, Star City, Moscow Region, Russia.; 13 Canary Center for Early Cancer Detection, Department of Radiology, Stanford University, Palo Alto, CA, USA.; 14 Institute for Regenerative Medicine, I.M. Sechenov First Moscow State Medical University, Moscow 119991, Russia.

## Abstract

Magnetic levitational bioassembly of three-dimensional (3D) tissue constructs represents a rapidly emerging scaffold- and label-free approach and alternative conceptual advance in tissue engineering. The magnetic bioassembler has been designed, developed, and certified for life space research. To the best of our knowledge, 3D tissue constructs have been biofabricated for the first time in space under microgravity from tissue spheroids consisting of human chondrocytes. Bioassembly and sequential tissue spheroid fusion presented a good agreement with developed predictive mathematical models and computer simulations. Tissue constructs demonstrated good viability and advanced stages of tissue spheroid fusion process. Thus, our data strongly suggest that scaffold-free formative biofabrication using magnetic fields is a feasible alternative to traditional scaffold-based approaches, hinting a new perspective avenue of research that could significantly advance tissue engineering. Magnetic levitational bioassembly in space can also advance space life science and space regenerative medicine.

## Teaser

Magnetic levitation is used for 3D biomanufacturing and biofabrication in space.

## INTRODUCTION

Traditional tissue engineering (TE) strategies are based on using biocompatible scaffolds for seeding cells; this concept was initially proposed by Langer and Vacanti (*1*). According to this concept, “scaffold” could be defined as a “temporal and removable support,” which makes it different from nonbiodegradable permanent implants and prostheses. Thus, the biodegradable scaffolds are considered as the most critical and essential element of TE technology framework that enables biofabrication of three-dimensional (3D) organ constructs and development of TE industry (*2*–*4*).

Apart from traditional scaffold-based TE, the novel “scaffold-free” approach, which does not require structural biomaterials, is being under investigation. Scaffold-free TE implies temporary supporting platforms that enable cell (or tissue spheroids) self-assembly or self-organization, proliferation, differentiation, and extracellular matrix (ECM) production (*5*–*7*). While some of them involve the use of different carrying temporal and removable support ranging from removable metallic needles (*8*) or removable agarose hydrogel (*9*), there are several strategies based on the living material self-assembly guided by field forces such as magnetic force (*10*–*12*), acoustic force (*13*, *14*), electrostatic force (*15*), and microgravity (*16*, *17*). In particular, principles of magnetic levitation first demonstrated by Geim’s group on living objects (*18*) and then successfully used in chemistry, material science, and biochemistry are also implied in TE (*19*, *20*). This approach uses magnetic levitation to guide the self-assembly of diamagnetic objects (cells or tissue spheroids) into 3D constructs in a paramagnetic fluid medium in the magnetic field gradient generated by strong magnets (*10*–*12*, *20*). In our previous study, we carried out the successful scaffold-free, label-free, and nozzle-free magnetic levitational bioassembly of tissue-engineered construct using chondrospheres as building blocks (*21*).

The choice of cartilage bioassembly in space experiments is determined, in particular, by a growing interest in the evaluation of effects of microgravity on human intervertebral discs and articular cartilages in the case of long-term spaceflights (*22*, *23*). So far, just two studies on biofabrication of cartilage in space have been performed, because space experiments are extremely expensive and time-consuming. In their seminal study, Freed *et al*. (*24*) grew bovine articular chondrocytes on polyglycolic acid scaffolds in rotating bioreactors for 3 months on Earth. After that, cartilaginous constructs were cultured aboard the Mir Space Station or on Earth for 4 months. In contrast, Stamenković *et al*. (*25*) grew porcine chondrocytes into cylindrical chambers under microgravity conditions on the International Space Station (ISS), under simulated microgravity conditions on a random positioning machine (RPM) and normal gravity for 16 days.

Despite the advantages of magnetic levitational bioassembly, there are problems related to the paramagnetic medium application. Gadolinium (Gd^3+^) chelates are the most commonly used paramagnetic agents. Although Gd^3+^-chelates are approved by the U.S. Food and Drug Administration as contrast agents for magnetic resonance imaging (MRI), Gd^3+^-chelates cause cytotoxicity and osmotic pressure imbalance in cells when used in high concentration (*26*, *27*). These adverse toxic effects of Gd^3+^ represent a challenge in advancing magnetic levitational bioassembly for basic and applied research. Theoretically, there are three possible ways to reduce undesirable toxic effects of paramagnetic medium: (i) develop low-toxic Gd^3+^-salts or alternative paramagnetic medium, (ii) perform levitational bioassembly in high magnet field, and (iii) perform magnetic levitational bioassembly under the conditions of microgravity. All these possibilities are subjects of ongoing systematic investigations.

**Fig. 1:**
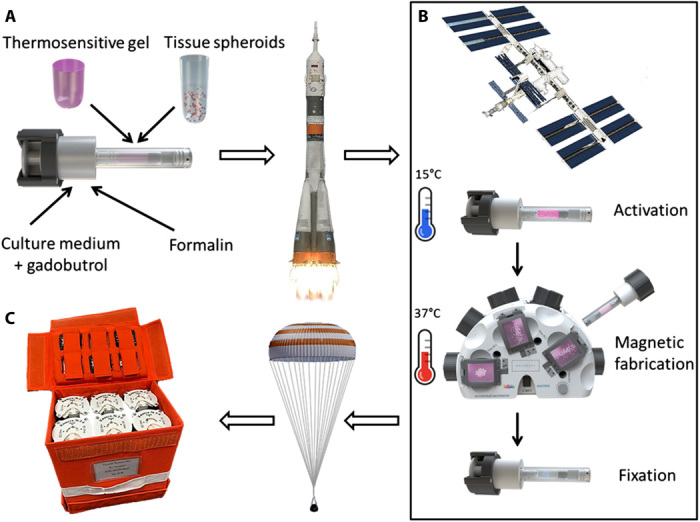
The schematic illustration of the space experiment. (**A**) Cuvettes filled with chondrospheres in thermoreversible nonadhesive hydrogel, culture medium with paramagnetic gadobutrol, and fixative solution (formalin). (**B**) Main stages of experiment performed on the ISS: activation of cuvettes by cooling down to 15°C, magnetic fabrication of 3D tissue constructs at 37°C, followed by fixation. (**C**) Transportation of cuvettes back to Earth. Photo credit: Vladislav A. Parfenov and Frederico DAS Pereira, Laboratory for Biotechnological Research “3D Bioprinting Solutions”, Moscow, Russia.

In the present study, we focus on levitational bioassembly of 3D cartilage construct under microgravity conditions in space. For biofabrication of cartilage construct aboard the ISS, we have developed a new technological approach involving a novel custom-designed magnetic bioassembler “Bioprinter Organ.Aut” (Fig. 1). Previously to space experiments, mathematical models and computer simulation for magnetic levitational assembly were developed to determine the kinetics of tissue spheroid fusion. The obtained 3D constructs in real space experiments were in close agreement with precalculated parameters. Thus, here, we report the results of the first-ever experiment devoted to magnetic levitational bioassembly at low, nontoxic Gd^3+^-chelate concentrations of tissue construct from living tissue spheroids in the condition of space microgravity.

## MATERIALS AND METHODS

### Experimental design

**Fig. 2:**
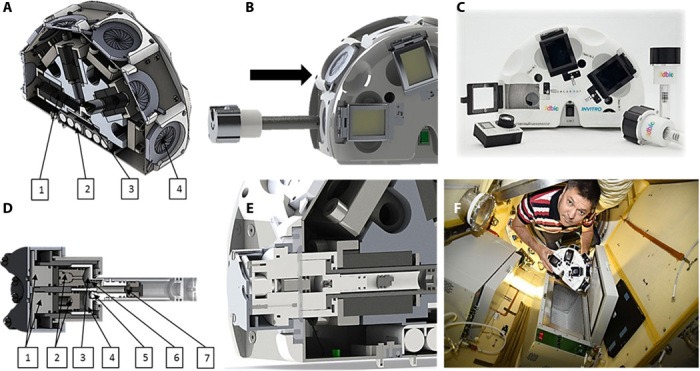
Scientific equipment of space experiment consisting of magnetic bioassembler Bioprinter Organ.Aut and hermetic cuvettes. (**A**) Cross section of magnetic bioassembler (1, magnets; 2, power sources; 3, light source; 4, one of the six cuvette ports). (**B**) Installation of the cuvette in the magnetic bioassembler. (**C**) Magnetic bioassembler and cuvettes. (**D**) Cross section of the cuvette (1, buttons-pistons for the injection of a nutrient medium and a clamp; 2, pistons of the secondary safety circuit; 3, volume for lock; 4, volume for the nutrient medium; 5, valve for the nutrient medium; 6, piston valve for the retainer; 7, volume for chondrospheres and thermoreversible hydrogel). (**E**) Cross section of cuvette inserted into magnetic bioassembler. (**F**) Russian cosmonaut Oleg Kononenko with the magnetic bioassembler and cuvettes. Photo credit: Vladislav A. Parfenov and Stanislav V. Petrov, Laboratory for Biotechnological Research “3D Bioprinting Solutions”, Moscow, Russia.

The experimental design of our study included several sequential steps (Fig. 1). Tissue spheroids (further referred to as “chondrospheres”) were fabricated from human chondrocytes on Earth in the biological laboratory located at the Baikonur Cosmodrome (Kazakhstan) and then placed into hermetically sealed cuvettes with admixed thermoreversible hydrogel (figs. S1 and S2), which prevented their undesirable preliminary fusion and attachment to the walls of cuvette during delivery to the ISS (Fig. 2). Charged cuvettes (fig. S3A) and custom-designed magnetic bioassembler Bioprinter Organ.Aut were delivered to the Russian segment of the ISS by the rocket “Soyuz-FG” and the spacecraft “Soyuz MS-11.” All sessions of magnetic levitational bioassembly were performed in the course of the Space Expedition “ISS 58-59” in December of 2018. At the start of the experiment, cosmonaut pushed the first button on the cuvettes (fig. S3B), then injected the paramagnetic medium into Mebiol gel with chondrospheres, and cooled it down to 17°C for 90 min in a temperature-controlled chamber, which provided the “gel-sol” phase transition of thermoreversible hydrogel (fig. S3C). This step released chondrospheres and enabled their free movement. After that, six cuvettes were simultaneously placed into magnetic bioassembler for 1 hour (fig. S3D) and then transferred into the temperature-controlled chamber (+37°C) for 2 days to sustain bioassembly and fusion of chondrospheres into tissue construct (fig. S3E). Video camera incorporated in magnetic bioassembler was used for recording the magnetic levitational bioassembly process (Fig. 2). Last, the obtained 3D tissue constructs were fixed in 4% formalin by pressing the second button on the cuvettes (fig. S3F) and then stored at room temperature for 2 weeks until return to Earth. The cuvettes with formalin-fixed 3D tissue constructs were landed by the spacecraft “Soyuz MS-09” and then subjected to detailed morphological and histological analysis.

During the experiments aboard the ISS, the objects were subjected to quasistatic accelerations up to 10^−6^ g, and harmonic vibrations in the frequency range from 5 to 20 Hz. The ambient climatic conditions were compensated by using hermetic cuvettes and a thermostatic chamber, maintaining the temperature range from +4° to +37°С at different stages of the experiment. When descending from the ISS, the object under study could experience shock pulses up to 67 g with a duration of 1 to 2 ms.

### Magnetic experimental setup

Magnetic bioassembler Bioprinter Organ.Aut (Fig. 2) and attached six cuvettes were custom-designed for space experiments to study 3D biofabrication of tissue-engineered constructs under microgravity condition. Each cuvette was intended to contain all necessary materials, providing biofabrication process, fixation, and retrieving of the biological samples. The cuvette comprises three volumes: a reservoir for culture medium with a capacity of 1 ± 0.2 ml, a reservoir for fixative solution with a capacity of 0.5 ± 0.1 ml, and a biofabrication chamber with a capacity of 2 ± 0.2 ml. The wall of the biofabrication chamber was transparent, allowing visual inspection and video recording of the experimental process. The two reservoirs were individually connected to the biofabrication chamber isolated by valves to avoid unwanted leakage of culture medium or fixative solution into the main chamber. The reservoirs for culture medium and fixative solution have their independent external plungers for being pressed by fingers. The valve installed in culture medium reservoir uses the Schrader valve principle, where the overpressure in the chamber (caused by pressing the external plunger) expands the rubber element, allowing the liquid to flow into the biofabrication chamber. The other valve unit installed in reservoir for fixation solution works like a gate valve, where the increase of pressure shifts a valve piston to the open position, clearing the orifice, allowing liquid transfer from a reservoir to biofabrication chamber. For biofabrication of 3D tissue constructs, the cuvettes with paramagnetic culture medium and chondrospheres had to be placed in the working zone of magnetic bioassembler (Fig. 1A), where the nonhomogeneous magnetic field was applied with a magnetic trap in the center. Magnetic bioassembler had six ports that allowed to install up to six cuvettes simultaneously at one space experiment. Three GoPro Hero4 action cameras (Woodman Labs Inc., USA) were used to perform video fixation of the biofabrication process.

The principle of experimental setup implicates the creation of a local minimum of magnetic field potential. Because of the nonhomogeneity of the magnetic field, the magnetophoretic force appears. It causes particle motion away from regions where the magnetic field is highly intensive. The magnetic force is applicable for particles with neutral charges and that have a relative permeability different from the background fluid. Then, the magnetic force **F**, acting on the object in the nonhomogeneous magnetic field, will be described by the following relation:

$$F=2\pi r^{3}\mu_{0}\mu_{f}K\nabla (H^{2})$$

where *H* is the magnetic field, *r* is the particle radius, μ_f_ is the relative fluid permeability, μ_p_ is the particle (spheroids) relative permeability, μ_0_ is the magnetic constant, and *K* is defined as:

$$K=\frac{\mu_{p}-\mu_{f}}{\mu_{p}+2\mu_{f}}$$

In our experiment, fluid and spheroid relative permeabilities are very close to 1, so magnetic force acting on particles is approximately linear with the difference between them. The difference μ_p_ − μ_f_ determines the direction of the magnetic force action. As a result, objects are pushed out into the area with lower field strength (magnetic trap) under the action of the magnetic force.

### Reagents

Agarose was purchased from Helicon (Russia, catalog no. Am-0710-0.1). Dulbecco’s modified Eagle’s medium (DMEM; catalog no. 12491-015), Dulbecco’s phosphate-buffered saline (PBS; catalog no. 18912-014), fetal bovine serum (FBS; catalog no. 16000-044), antibiotic-antimycotic (catalog no. 15240-062), and trypsin/EDTA (catalog no. 25200-114) were obtained from Gibco (USA). l-Glutamine (catalog no. F032) was obtained from Paneco (Russia). Paraformaldehyde (catalog no. P6148-500G) and resazurin sodium salt (catalog no. R7017-5G) were obtained from Sigma-Aldrich (USA). Gadovist (gadobutrol) was obtained from Bayer (Germany). “Mebiol” gel (catalog no. MBG-PMW20-5001) was obtained from Cosmo Bio (Japan). CellTiter-Glo 3D Cell Viability Assay (catalog no. G9682) was purchased from Promega (USA).

### Cell culture

Human chondrocytes were purchased from PromoCell (catalog no. C-12710) and grown in DMEM containing 10% FBS and 2 mM l-glutamine and supplemented with penicillin (100 U/ml), streptomycin (100 μg/ml), and amphotericin B (250 ng/ml). The cells were incubated at 37°C in a humidified atmosphere with 5% CO_2_ and routinely split at 85 to 95% confluence. Cell transfer and preparation of cell suspensions were performed using mild enzymatic dissociation with a 0.25% trypsin/0.53 mM EDTA solution. The cells of P4 to P6 passages were used in all experiments. During cultivation, their morphology and proliferation were observed using inverted phase-contrast microscopy (fig. S4).

### Formation of chondrospheres using MicroTissues 3D Petri dishes

The chondrospheres were formed using MicroTissues 3D Petri dish micro-molds (Sigma-Aldrich, catalog no. Z764019-6EA, 81 circular wells 800 μm × 800 μm) as previously described (*28*). The concentration of human chondrocytes was 3.4 × 10^6^ per milliliter, which resulted in a final concentration of 8000 cells per well/spheroid. The chondrospheres were incubated at 37°C in a humidified atmosphere with 5% CO_2_ for 2 days. On the second day of fabrication, the diameters and roundness of chondrospheres were estimated using bright-field microscopy (Nikon Eclipse T*i*-E inverted microscope, Japan) and measured using ImageJ 1.48v software [National Institutes of Health (NIH), Bethesda, MD, USA] as previously described (*28*).

### Biomechanical testing

The biomechanical properties of chondrospheres were measured with a microscale parallel-plate compression testing system (MicroSquisher, CellScale, Canada) and associated SquisherJoy software. The chondrospheres were formed using Corning spheroid microplates (Corning, catalog no. 4520) according to the manufacturer’s protocol at the final concentrations of 8000 cells per chondrosphere. Corning spheroid microplates were incubated at 37°C in a humidified atmosphere with 5% CO_2_ for 2 days. Then, 2-day-old chondrospheres were placed in Mebiol gel (Cosmo Bio, catalog no. MBG-PMW20-5001) for 72 hours. Control chondrospheres were incubated in complete culture medium at 37°C in a humidified atmosphere with 5% CO_2_. For biomechanical testing, chondrospheres were placed in a PBS-filled bath at 37°C and compressed to 50% deformation in 20 s. The microbeam with a diameter of 304.8 μm (recommended maximal force of 917 mN) was used. The force-displacement data obtained from the compression test were converted to stress-strain curves, and the lower portion of the curve (0 to 20% strain) was used to obtain a linear regression line and to estimate the elastic modulus. Twenty samples of chondrospheres were measured in each group.

### Cell viability assay

Human chondrocytes were seeded in 96-well culture plate at a concentration of 1 × 10^4^ cells per well. Each well contained 100 μl of cell suspension. The plate was incubated for 24 hours at 37°C in a humidified atmosphere with 5% CO_2_ to obtain a premonolayer culture. Then, the different concentrations of gadobutrol were added to experimental wells and the plate was incubated for additional 24 hours at 37°C in a humidified atmosphere with 5% CO_2_, following which 0.02% resazurin solution in culture medium was added to each well of plate. The plate was returned to CO_2_ incubator for 6 hours, and then fluorescence was recorded at excitation wavelength of 555 nm with emission detected at 580 nm using the VICTOR X3 Multilabel Plate Reader (PerkinElmer, USA). The wells containing cell culture medium without any cells were used to assess background signal.

### Nonadhesive hydrogel and medium for magnetic levitational bioassembly

Ultimate complete medium for magnetic levitational bioassembly consisted of DMEM supplemented with l-glutamine, 10% FBS, and 10 mM gadobutrol. Gadobutrol (C_18_H_31_GdN_4_O_9_) is a nonionic, paramagnetic complex consisting of gadolinium (Gd^3+^) chelated with the macrocyclic dihydroxy-hydroxymethylpropyl-tetraazacyclododecane-triacetic acid (butrol). At the beginning of the space experiment, each cuvette contained 600 μl of thermoreversible Mebiol hydrogel with entrapped 180 chondrospheres. After cooling and phase transition to liquid sol, it was diluted with 1 ml of complete medium, then thoroughly mixed by turning in hands and shaking, and finally installed in the port of «Bioprinter Organ.Aut» for magnetic levitational bioassembly. Six 3D tissue constructs in six cuvettes were biofabricated during space experiments.

### Estimation of transporting thermoreversible hydrogel effects on chondrosphere viability and fusion capability

To enable the safe delivery of viable chondrospheres from Earth to space, avoiding their fusion and spreading in cuvettes, we used commercially available nonadhesive PNIIPAAm-PEG [copolymer of poly(N-isopropylacrylamide) and poly(ethylene glycol)] thermoreversible hydrogel of the Mebiol brand (Cosmo Bio, Japan). We evaluated the potential toxic effect of Mebiol gel on chondrospheres because it was to be embedded in hydrogel for several days during delivery to the ISS. Two-day-old chondrospheres were placed in hydrogel at room temperature for 72 hours. Control chondrospheres were incubated in complete culture medium at 37°C in a humidified atmosphere with 5% CO_2_. The viability of chondrospheres was assessed using the CellTiter-Glo 3D Kit according to the manufacturer protocol. The CellTiter-Glo 3D Kit was added, and luminescence was recorded after 60 min of incubation using the VICTOR X3 Multilabel Plate Reader (PerkinElmer, USA). Qualitative assessment of cell viability within chondrospheres was performed using the Live/Dead Cell Double Staining Kit (Sigma-Aldrich, USA) according to the manufacturer’s protocol. Chondrospheres were incubated in the solution containing calcein AM and propidium iodide at 37°C for 1 hour. After washing with PBS, spheroids were imaged with a fluorescent microscope (Nikon Eclipse T*i*-E, Japan).

Spheroid fusion assay was performed using nonadhesive Corning spheroid microplates (Corning, catalog no. 4520). Pairs of chondrospheres were placed in one well and incubated for 48 hours. Bright-field images of chondrosphere doublets were obtained at points 0, 1, 2, 4, 6, 24, and 48 hours using a “Nikon Eclipse T*i*-E” microscope. Intersphere angle was measured using “ImageJ 1.48v” software (NIH, Bethesda, MD, USA) and plotted as a function of time using “GraphPad Prism” software (GraphPad Software Inc., La Jolla, CA).

### Rheological testing of thermoreversible hydrogel

Rheological measurements were performed under oscillatory shear mode using a rotational Anton-Paar MCR 501 rheometer. Samples of pure Mebiol solution and work solution were placed between cone (CP50-1/TG-SN15826; *d* = 0.05 mm) and plate with a diameter of 50 mm and a gap of 0.5 mm. After cooling to +4°C, the edges were covered with mineral oil to prevent dehydration. The temperature dependence of storage modulus (*G*′), loss modulus (*G*″), and complex viscosity (η*) was measured using a temperature sweep (oscillation) by increasing the temperature from +4° to +37°C at a heating rate of +5°C min^−1^. The frequency and shear strain were maintained constant at 10 rad s^−1^ and 1%, respectively. Twenty samples were measured in each group.

### Computer simulation of the magnetic field

The 3D nonhomogeneous static magnetic field was modeled using multiphysics computational program COMSOL by the finite element method. Magnetic field was calculated according to material grade of NdFeB magnet N38 (Residual magnetic induction - 1.21 T), and magnetic properties of paramagnetic medium were chosen equal to experimental ones: They changed depending on gadobutrol concentration. The simulation of the magnetic field allowed calculating the particle trajectory equation. The transient calculation of particle trajectories was conducted using COMSOL “Particle Tracing Module.” During this calculation, the following forces were taken into account: Magnetic force based on the difference between medium and particle magnetic permeabilities, drag force affecting the time of assembly, and the elastic force of particle-particle interaction. Because of low velocities of particles, the Stokes’ drag law was used to describe viscous resistance. Physical characteristics of particles were chosen in correspondence with a proposed experiment: The particle diameter was 0.2 mm, its density was taken as 1050 kg m^−3^, the shape of the particles was assumed to be spherical, and the total number of simulated particles was 400. The features of paramagnetic liquid were found experimentally and proved to be as follows: Density was around 1000 kg m^−3^ and dynamic viscosity η was 0.0155, 0.0474, and 2.49 Pa·s at 8°, 17°, and 37°C, respectively. As can be seen, the value of dynamic viscosity depended only on temperature and was equal for different concentrations of gadobutrol (50, 10, and 0.8 mM). The paramagnetic permeability of liquid was 1.0000054, 1.0000011, and 0.000000087 for 50, 10, and 0.8 mM gadobutrol, respectively. The calculated velocities and trajectories of particles corresponded well to the experimental data.

### Simulation of chondrosphere fusion process

The process of chondrospheres fusion was modeled using the open-source software Surface Evolver (*29*). Chondrospheres were approximated and modeled as ball-like liquid droplets of standard size and volume. Initially, the neighboring chondrospheres were located at random. The process of fusion of chondrospheres was modeled by iterating gradient descent to minimize the surface energy subject to the constraint of constant spheroid volumes until movement stopped. The evolution script created interfaces between chondrospheres where chondrospheres touched. The configuration reached a local minimum of energy but not necessarily a global minimum. The progressive changes in the shape of single chondrospheres contacted with each other inside forming compacted tissue constructs have been visualized. The simulations have been performed with 40 chondrospheres.

### Transmission electron microscopy

For transmission electron microscopic (TEM) examination, chondrospheres, native or exposed to 10 or 50 mM gadobutrol, were fixed in a 2.5% solution of glutaraldehyde in 0.1 M phosphate buffer, then treated with 1% OsO_4_ solution in 0.1 M phosphate buffer, and dehydrated in descending alcohols and finally in propylene oxide. After dehydration, chondrospheres were placed in a mixture of propylene oxide and araldite and then into araldite. Semi-thin and ultrathin sections were obtained using ultramicrotome Leica EM UC7 (Leica, Germany). Ultrathin sections were obtained using an LKB-III ultramicrotome (LKB, Sweden) and then contrasted with 4% (w/v) water solution of uranyl acetate (40 min at 37°C) and 0.04% (w/v) water solution of lead citrate (Reynolds 1963) for 20 min at room temperature. The sections were examined under a JEM 100CX-II TEM (JEOL, Japan) equipped with an ES400W Erlangshen digital camera (Gatan, United Kingdom) using an accelerating voltage of 80 kW. Semi-thin sections were cut using LKB-III ultramicrotome (LKB, Sweden), stained with 1% toluidine blue, and viewed in a Leica DM 2500 light microscope equipped with a Leica DFC 290 digital camera (Leica, Germany).

### Histology and immunohistochemistry

The formalin-fixed samples were put in custom-made agarose molds for stereo microscopy evaluation (Nikon SMZ18, Japan) and then embedded in paraffin (BioVitrum, Russia). Serial sections with a thickness of 4 μm were cut with Microtome HMS 740 (Thermo Fisher Scientific, USA) and mounted on poly-l-lysine–coated glass. Dewaxing was carried out in xylene and a battery of downstream alcohols, and antigen retrieval was performed using proteinase K (Sigma-Aldrich, USA) for 20 min. After that, samples were incubated in peroxidase-blocking solution for 10 min in protein-blocking solution for 10 min followed by incubation with primary antibodies for 30 min, incubation with secondary antibody for 10 min, and 5-min 3,3′-diaminobenzidine (DAB) treatment. The above procedures were performed automatically in an Autostainer 360 system (Thermo Fisher Scientific, USA). Washing was carried out in tris buffer (pH 6.0) with Tween 20. Primary polyclonal rabbit antibodies to human caspase-3 (Abbiotec, USA) were used to reveal the apoptosis inside the biofabricated construct. Primary antibodies to type II collagen followed by 30-min incubation with biotinylated secondary antibody (both Novocastra, Leica Microsystems) were used for visualization of ECM production in chondrospheres. Nuclei were counterstained with Mayer’s hematoxylin. Last, sections were dehydrated and enclosed in Bio-Mount (Bio Optica Milano S.P.A., Italy). Some sections were routinely stained with hematoxylin and eosin (BioVitrum, Russia) and then examined by light microscopy (Nikon Eclipse T*i*-E, Japan). For the visualization of glycosaminoglycans, fixed spheroids were washed with PBS and stained overnight with Alcian blue solution (1% in 0.1 N HCl). The results were examined using AxioVert 2 inverted light microscope (Zeiss) equipped with a Nikon D610 DSLR camera (Nikon).

### Statistics analysis

Statistical data were analyzed using GraphPad Prism software (GraphPad Software Inc., La Jolla, CA) and represented as means ± SEM. The analysis of variance (ANOVA) test was used to find the notable differences between the means of the three groups with *P* < 0.0001.

## RESULTS

### Mathematical modeling of the magnetic field and computer simulation of construct assembly

**Fig. 3:**
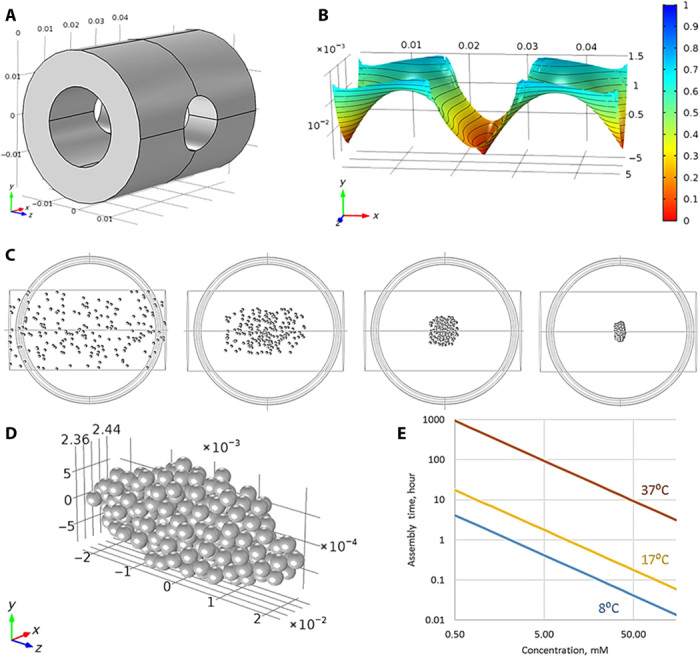
Simulation of the magnetic field and kinetics of tissue spheroid assembly. (**A**) System of magnets installed into magnetic bioassembler. (**B**) Magnetic field generated by system of magnets. (**C**) Modeling of construct assembly process. (**D**) Modeled shape of the construct after assembly. (**E**) Kinetics of the construct assembly as a function of gadobutrol concentrations and temperature.

The magnetic field was initially modeled using COMSOL software (Fig. 3A). An example of the *y* component of the magnetic field at *xz* plane is shown in Fig. 3B. The magnetic trap location corresponded with the center of setup. It had been also shown that the most efficient way to assemble spheroids was to put them inside the short tube in the center of the magnetic field because, outside of the central in-magnet area, the magnetic force pushed particles to the side regions.

The simulation also allowed the evaluation of the assembly kinetics (Fig. 3C and movies S1 to S4) and the time of full assembly. It was found that assembly time strongly depended on paramagnetic medium viscosity and its magnetic permeability. The results of computer simulation and experiment data demonstrated a good correspondence between theoretical and empiric assembly times at 10 mM concentration of gadobutrol at the temperature of 17°C.

However, numerical simulation demands quite a long time to conduct calculation of a single case, so we developed the theoretical model to predict the dependence of assembly time on parameters of the experiment. We assumed that there were magnetic and shear forces acting on spheroids. Because of low velocities of movement, the shear force was described by Stokes law. Then, we could write the equation of particle motion and estimate time of assembly. This time depended on the initial position of each particle; therefore, in general case, we could find a function that described the duration of assembly with accuracy up to a constant (fig. S4), and this constant was the same for similar particle positions.

The resulting equation for assembly time was as follows:

$$T_{\text{ass}}\sim 3\eta L/4r^{2}\mu_{0}\mu_{f}{\mathrm{KH}}_{\text{max}}^{2}$$

where *L* is a width of permanent magnet and *H*_max_ is the maximum value of the magnetic field inside magnets. From this equation, it is evident that assembly time is proportional to the relation of fluid viscosity η to difference of magnetic permeability μ_f_ − μ_p_, which is linear with gadobutrol concentration. Thus, knowing the exact assembly time for a specific gadobutrol concentration in the medium, we could accurately estimate the assembly time of the same system with a different gadobutrol concentration. It was clear that, when the *T*_ass_ value was a few seconds or minutes, we could talk about the assembly of particles into a construct, and when the *T*_ass_ value was several hours or even days, the assembly process would be too slow. It was critical in the case of chondrospheres because viable particles could be damaged by a loss of oxygen and nutrients and become unusable faster than merge into a construct.

The dependence of the assembly time on gadobutrol concentration was also calculated (Fig. 3E). The resulting function correlated with experimental data at a concentration of 10 mM (~40 min). Moreover, it was possible to estimate the time of assembly at a minimal concentration of 0.8 mM—it would be 10 hours according to mathematical calculations. However, as can be seen from the curve (Fig. 3E), the reduction of temperature during the assembly could theoretically accelerate the process.

### Rheological properties of thermoreversible hydrogel

Temperature sweep (oscillation) was conducted by heating of pure or diluted PNIPAAm-PEG from 4° to 37°C with a heating rate of +5°C min^−1^ (fig. S5). For both variants, “sol-gel” transduction occurred, but unlike from undiluted hydrogel composition in which the temperature of sol-gel transduction was 16.5° ± 0.5°C, the temperature of transition for 1.6 times diluted work solution increased to 27.1° ± 0.7°C. As sample temperature raised to the gelation state, both *G*′ and *G*″ rapidly increased due to the formation of a hydrogel. It is worth to note that the *G*′ and *G*″ values at 37°C reached 903 ± 37 Pa and 206 ± 19 Pa for undiluted hydrogel solution and 6.3 ± 1.2 Pa and 2.3 ± 0.6 Pa for work solution. Viscosity measurements also showed a similar trend. At 4°C, the values of complex viscosity of undiluted hydrogel solution and work solution were 0.211 ± 0.05 Pa·s and 0.97 ± 0.04 Pa·s, respectively. Moreover, at 37°C, the values of complex viscosity of undiluted hydrogel solution and work solution were 936 ± 21 Pa·s and 6.77 ± 0.53 Pa·s, respectively. Thus, the rheological properties of hydrogel diluted with complete medium provided favorable conditions for magnetic bioassembly and subsequent fusion of 3D tissue construct.

### Characterization of chondrospheres

**Fig. 4:**
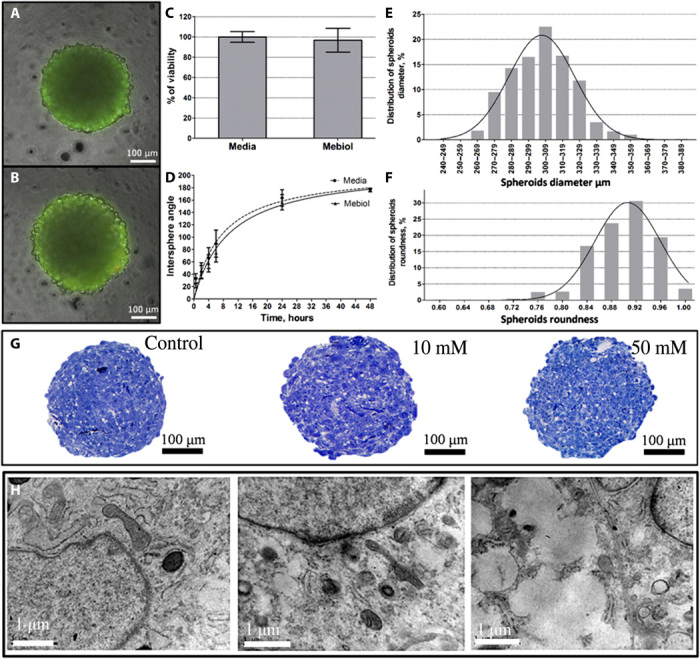
Evaluation of biofabrication medium toxicity and rheological parameters. (**A**) Chondrosphere after 72-hour incubation in Mebiol gel. (**B**) Chondrosphere after 72-hour incubation in culture medium (control) (Live/Dead assay, living cells are stained green). (**C**) Quantitative assessment of viability of chondrospheres after 72-hour incubation in Mebiol gel and culture medium by using the CellTiter-Glo Kit. (**D**) Time-curve of intersphere angles for pairs of chondrospheres during the fusion. (**E**) Distribution of diameters of 2-day-old chondrospheres. (**F**) Roundness of 2-day-old chondrospheres. (**G**) Chondrospheres without or after exposure to 10 mM and 50 mM gadobutrol for 24 hours; toluidine blue staining. (**H**) TEM images of cells within chondrospheres without or after exposure to 10 mM and 50 mM gadobutrol. Some of the representative data are shown. Photo credit: Elizaveta Koudan, Laboratory for Biotechnological Research “3D Bioprinting Solutions”, Moscow, Russia.

Chondrospheres were made from characterized human chondrocytes (fig. S3) using agarose micro-molds. For estimation of the PNIPAAm-PEG thermoreversible hydrogel cytotoxicity, 2-day-old сhondrospheres were incubated in hydrogel at room temperature for 72 hours. Then, their viability was evaluated using the Live/Dead Cell Double Staining Kit (Fig. 4, A and B) and the CellTiter-Glo 3D Kit (Fig. 4C). As a result, cell viability was 97 ± 6%. The biomechanical properties (Young’s modulus) of chondrospheres were measured using micro-scale tensiometry and also revealed no differences between control and hydrogel-treated samples (fig. S7). Thus, the thermoreversible hydrogel was considered nontoxic for cells and acceptable for tissue spheroid transportation.

The application of chondrospheres as building blocks requires the standardization of their size and shape (*30*). In this term, we measured spheroid diameters and roundness of 2-day-old сhondrospheres (Fig. 4, D to F). The average diameter was 300 ± 13 μm. The average spheroid roundness was 0.91 ± 0.05. Little variance indicated that chondrospheres had uniform standardized morphology and could be used for biofabrication of 3D tissue constructs with precise geometry.

Another parameter that could affect the viability and physiology of chondrospheres was gadobutrol (Gd^3+^-chelate)—the main component of the paramagnetic medium provided the magnetic levitation in our settings. Different methods assessed possible toxic effects. The histological and histochemical study demonstrated the absence of significant apoptosis and cell death inside the spheroids at 10 mM gadobutrol (Fig. 4G), while high concentration (50 mM) caused some softening and loss of roundness in chondrospheres. According to TEM data, most of the cells preserve intact normal ultrastructure in tissue spheroids at low concentration (10 mM) of gadobutrol. In contrast, at 50 mM, some indications of intracellular dystrophy, i.e., margination of chromatin, swelling of mitochondria and endoplasmic reticulum, accumulation of phagosomes, and multiple intracellular membrane vacuoles, were observed (Fig. 4H).

### Magnetic levitational bioassembly of 3D tissue constructs in space

**Fig. 5:**
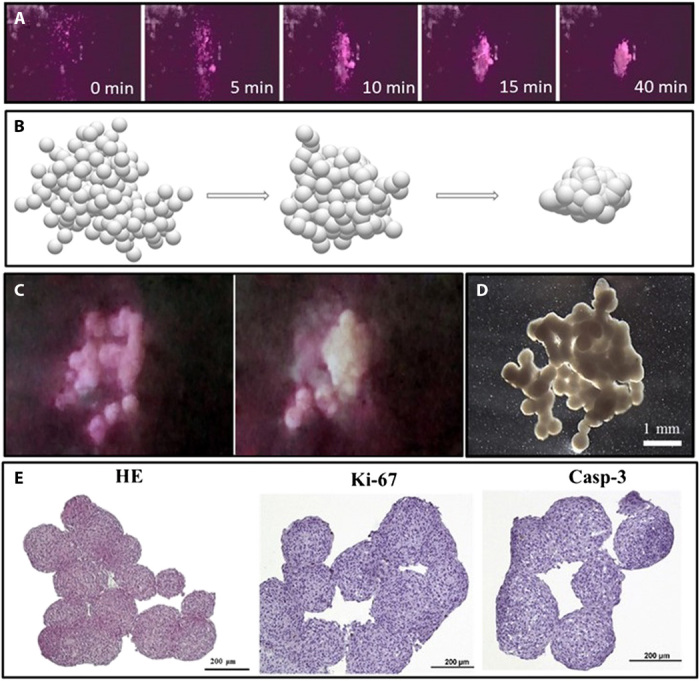
Morphological studies of 3D tissue construct obtained by magnetic levitation in space. (**A**) Time-lapse photographs of the construct assembly inside the magnetic bioassembler in space. (**B**) Computer simulation of chondrosphere fusion into 3D construct using “Surface Evolver” software. (**C**) Real sequential steps of construct bioassembly in space; snapshots from time-lapse video recording. (**D**) Macrophotography of assembled 3D construct returned to Earth. (**E**) Histology [hematoxylin and eosin (HE) staining] and immunohistochemistry [proliferation marker Ki-67 and apoptosis marker caspase-3 (Casp-3)] of 3D tissue construct assembled in space experiment. Photo credit: Kenn Brakke, Susquehanna University, Selinsgrove, PA, USA; Elizaveta Koudan, Laboratory for Biotechnological Research “3D Bioprinting Solutions”, Moscow, Russia.

The developed experimental setup provided the biofabrication experiment in space aboard the ISS. Figure 5A and movie S5 demonstrate the consecutive assembly of construct from tissue spheroids. The performance and duration of the assembly process were in good agreement with the predictive model (Fig. 3E). The used «Surface Evolver» software (*29*) allowed visualization of the sequential process of tissue spheroid fusion (Fig. 5B). The experiment with magnetic levitation bioassembly of 3D tissue constructs on the ISS was carried out in two steps. First, the configuration of the magnetic field in the bioassembler provided tissue spheroid movement to local area and formation of multiple spatial contacts. This stage was performed at ambient temperature aboard the ISS and took 40 min at 10 mM concentration of gadobutrol in paramagnetic medium. These assembly parameters were selected according to mathematical modeling to maintain the maximum viability of spheroids necessary for subsequent fusion. At the end of this stage, tissue spheroids were tightly packed but still did not have tight junctions between cells on the surface of the spheroids (Fig. 5C). In the second stage, which lasted 48 hours at 37°C, tissue spheroids started to fuse and form stable 3D tissue construct (Fig. 5D). A comparison of the real image with computer simulation indicated that obtained constructs represented advanced stages of tissue spheroid fusion. According to mathematical modeling, the level of tissue spheroid fusion completeness was higher than 50%, and in some fragments, it achieved more than 90% of possible compaction. Taking this into account, we could assume that the elongation of biofabrication time would enable the complete fusion of chondrospheres into a single 3D tissue construct.

The histological analysis also revealed the different kinetic of chondrospheres inside the construct. As a result, there were some gaps or incomplete fusion in tissue that reflects random initial packing of chondrospheres (Fig. 5E). However, specific immunohistochemistry staining revealed no features of apoptosis (caspase-3 expression) inside the construct. Cells did not proliferate during the fusion of chondrospheres that was shown by the almost absent expression of the Ki-67 marker. Thus, the selected and tested conditions of delivery and subsequent assembly of tissue constructs in space allowed the chondrocytes to maintain their viability and physiological activity typical for cellular behavior in 3D cultures.

## DISCUSSION

The development of modern technologies for deep space exploration and the extension of manned space capabilities steadily increase the importance of space biotechnologies. So, even today, it has become possible to grow aboard the ISS different species of plants producing oxygen and nutrients (*31*), to obtain 3D biofilms of bacteria with altered synthetic and physiological activity (*32*), and to grow large protein crystals with an isometric internal structure to develop new anticancer or antiviral drugs (*33*, *34*). In this regard, the development of TE approaches to create complete equivalents of human tissues and organs to study the influence of space flight conditions and to meet the needs of space medicine is the next consequential step.

Because space experiments are expensive and rare, several devices are frequently used to simulate microgravity on Earth—fast-rotating clinostat, free-fall machine, RPM, and rotating wall vessel (RWV) (*16*, *17*, *35*). The RPM has been shown to reproduce microgravity conditions correctly for leukocytes and human leukemic monocyte/macrophage cell line U937 (*35*–*37*). In two studies, the results from RPM experiments were directly compared to results from simultaneously performed space experiments (*25*, *38*). RPM has been shown to mimic several cellular responses associated with microgravity, but not all (*39*). RPM and RWV have been successfully used to study the rearrangement of the cytoskeleton in cells cultured in a monolayer or suspension, the altered activity of mechanosensitive ion channels, as well as the changes in intracellular signal transmission and following gene expression in the absence of gravitational effects (*39*–*41*). Moreover, because simulated microgravity provides aggregation and tissue-specific differentiation of cells (*42*), RPM and RWV have been used to grow tissue equivalents (*24*, *25*, *43*). Nevertheless, simulated microgravity on Earth does not reproduce exactly the real space environment (*43*).

Transportation of viable tissue spheroids from Earth to the ISS and prevention of their undesirable preliminary tissue fusion during transport was one of the main challenges in this project. It was achieved by using biocompatible PNIPAAm-PEG thermoreversible hydrogel, which represents gel at physiological temperature 37°C and transforms into sol at a temperature below 21°C. Our data demonstrated that tissue spheroids could survive for up to 7 days being embedded in thermoreversible hydrogel inside the closed cuvette systems. In our current space study, we used chondrospheres that are capable of maintaining their viability in a state of hypoxia for a rather long time as well as the ability to produce ECM components (*28*, *44*). It is also crucial that chondrocytes are very resistant to stress caused by microgravity (actual or simulated) (*26*, *45*). Alternative approaches such as fabricating tissue spheroids from cell monolayers labeled with magnetic nanoparticles (*46*) or spontaneous formation of tissue spheroids from monolayer in space (*40*) currently do not provide a sufficient number of tissue spheroids with standard size and shape for reproducible experiments. At the same time, the shift to biofabrication of more metabolically active tissue structures, i.e., from epithelial, muscle, or nerve cells, apparently will require the implementation of additional perfusion bioreactors that provide a continuous supply of oxygen and nutrients during the experiment (*47*, *48*). However, the application of such systems on ISS requires special equipment and cell culture skills among the astronauts. At the same time, we can assume the possibility of developing systems with artificial gravity (~1*g*) for the formation of standardized tissue spheroids aboard the space stations in the future.

The main reason for us to make the described experiment in space was the assumption that microgravity would give us the possibility to perform magnetic levitation at a low, nontoxic concentration of paramagnetic Gd^3+^-chelates in comparison with ground conditions. Tissue spheroids are rather large and heavy biological objects, so at a preliminary stage of the current study, we faced a dilemma—using paramagnetic medium, from one side, is critically important for enabling magnetic levitation; from another side, the toxic effects of Gd^3+^ are well known and were previously reported (*26*, *27*, *45*). According to our previous TEM analysis, 50 mM gadobutrol provides effective magnetic levitation of chondrospheres in a laboratory setup on Earth but causes changes in cellular ultrastructure: margination of nuclear chromatin, swelling of mitochondria and endoplasmic reticulum, accumulation of phagosome, and multiple membrane vacuoles, which are considered as Gd^3+^-induced intracellular dystrophy (*21*). In space experiment, we used 10 mM gadobutrol, which does not affect cell viability crucial for tissue spheroid fusion into 3D construct (fig. S8). In routine clinical practice, the dose of administered gadobutrol for MRI provides a plasma concentration of 0.3 to 0.6 mM. Taking into account the known affinity of Gd^3+^ to calcium channels, we can hypothesize the possibility of developing reversible metabolic changes in organ constructs obtained from muscle or other Ca^2+^-dependent cells that will require the development of effective protocols for its removal from constructs after completion of the magnetic bioassembly process.

Lower concentrations of paramagnetic could also contribute to better survival of organ constructs obtained by magnetic levitation in our setting. However, this option also has its limitations, because a decrease in the concentration of gadobutrol will increase the assembly time of the construct in adverse temperature conditions. If the assembly time of less than 1 hour is considered acceptable, then a particular minimum concentration of the paramagnetic could be determined. Because in our setting a diluted thermoreversible hydrogel remains in the final solution of cuvettes, the viscosity of such medium and, consequently, the assembly rate also strongly depend on the temperature. According to our mathematical calculations, the concentration of gadobutrol in this system could be, theoretically, reduced up to 0.8 to 1.0 mM, provided that the biofabrication will occur at a temperature of 4°C. The assembly time, in this case, will be approximately 30 min, which allowed carrying out of experiment without affecting tissue spheroid viability. Verification of these conditions should be the subject of research in future space experiments.

Magnetic bioassembler Bioprinter Organ.Aut used in the current study represents a new research tool intended and adopted for magnetic levitational experiments on the ISS. It is a first equipment that allows us to perform the magnetic levitational biofabrication experiments under microgravity conditions in space in a reliable closed system. Despite its compactness, magnetic bioassembler allows us to simultaneously perform six experiments, providing an opportunity to get reproducible and statistically significant data. Video cameras incorporated into magnetic bioassembler allow registration of tissue construct assembly process during the experiments. The magnetic bioassembler will remain on the Russian segment of the ISS for at least 5 years, giving the possibility for future experiments. Thus, the experiments in biofabrication of 3D constructs based on other cell types can be further performed without the need to deliver the whole system, which will substantially reduce the shipping cost. With a certain level of automation and development of additional biosensor, such a system can be upgraded for the man-free study of space radiation effects on human tissues. In the case of developing a fully automated system for real-time biomonitoring, the final return of specimens to Earth will not be obligatory. In out next-coming experiments, we are going to modify the cuvette with ultrasonic transducers to apply combined magnetic and acoustic fields for the biofabrication of structures with more complex geometry, i.e., tubular constructs of trachea or urethra.

The technology of magnetic levitation in space will also make it possible to obtain combined constructs from biological and inorganic materials, such as bone tissue equivalents consisting of tissue spheroids and granules of calcium phosphates. Because of significant differences in the densities of these materials, obtaining these constructs by magnetic levitation assembly in Earth conditions, even using superconducting or Bitter magnets, is extremely difficult due to the considerable differences in the necessary intensities of the magnetic fields that will ensure their levitation. The possibility of obtaining hybrid constructs of complex shape, consisting of living and nonliving building blocks, will give a powerful impetus to the development of biofabrication in space and also should be the subject of future research. Thus, the magnetic levitational bioassembly of viable tissue-engineered constructs reported here had been realized for the first time in space microgravity conditions using practically nontoxic concentration of Gd^3+^-salt.

## CONCLUSION

Scaffold-free, label-free, and nozzle-free magnetic levitational bioassembly of 3D tissue constructs from tissue spheroids (chondrospheres) has been performed for the first time at the condition of real microgravity in space. It has been demonstrated that nonadhesive biocompatible and thermo-reversible hydrogel enables the delivery of viable tissue spheroids to the ISS and prevents their undesirable preliminary tissue fusion and spreading. The novel original magnetic bioassembler has been designed, implemented, certified, and successfully tested for space biotechnology research. Magnetic levitational bioassembly of viable tissue construct has been accomplished in magnetic bioassembler at a nontoxic concentration of paramagnetic–Gd^3+^-chelate gadobutrol. Assembly and fusion of tissue construct have been realized in good agreement with developed predictive mathematical models and computer simulations. The magnetic levitational bioassembly of 3D tissue construct from tissue spheroids in microgravity at a nontoxic concentration of paramagnetic medium is an important milestone in the advancing of the new approach. We strongly believe that it opens a new avenue for research in the evolving formative biofabrication field.

## Supplementary Material

aba4174_Movie_S5.mp4

aba4174_Movie_S4.mp4

aba4174_Movie_S6.mp4

aba4174_Movie_S3.mp4

aba4174_Movie_S2.mp4

aba4174_SM.pdf

aba4174_Movie_S1.mp4

## SUPPLEMENTARY MATERIALS

Supplementary material for this article is available at http://advances.sciencemag.org/cgi/content/full/6/29/eaba4174/DC1

## Appendix

View/request a protocol for this paper from *Bio-protocol*.
