# Supplementary material for: Magnetic levitational bioassembly of 3D tissue construct in space
Source: Sci Adv. 2020 Jul 15;6(29):eaba4174. doi: 10.1126/sciadv.aba4174 (PMC7363443; doi:10.1126/sciadv.aba4174)
Supplement: aba4174_SM.pdf [file aba4174_SM.pdf]

Science Advances
AAAS

advances.sciencemag.org/cgi/content/full/6/29/eaba4174/DC1

Supplementary Materials for

Magnetic levitational bioassembly of 3D tissue construct in space

Vladislav A. Parfenov*, Yusef D. Khesuani, Stanislav V. Petrov, Pavel A. Karalkin, Elizaveta V. Koudan, Elizaveta K. Nezhurina, Frederico DAS Pereira, Alisa A. Krokhmal, Anna A. Gryadunova, Elena A. Bulanova, Igor V. Vakhrushev, Igor I. Babichenko, Vladimir Kasyanov, Oleg F. Petrov, Mikhail M. Vasiliev, Kenn Brakke, Sergei I. Belousov, Timofei E. Grigoriev, Egor O. Osidak, Ekaterina I. Rossiyskaya, Ludmila B. Buravkova, Oleg D. Kononenko, Utkan Demirci*, Vladimir A. Mironov*

*Corresponding author. Email: vapar@mail.ru (V.A.P.); vladimir.mironov54@gmail.com (V.A.M.); utkan@stanford.edu (U.D.)

Published 15 July 2020, *Sci. Adv.* **6**, eaba4174 (2020)
DOI: 10.1126/sciadv.aba4174

The PDF file includes:

Sections S1 to S3
Figs. S1 to S8
Legends for movies S1 to S6

Other Supplementary Material for this manuscript includes the following:

(available at advances.sciencemag.org/cgi/content/full/6/29/eaba4174/DC1)

Movies S1 to S6

Magnetic levitational bioassembly of 3D tissue construct in Space microgravity at a non-toxic concentration of paramagnetic medium has been performed first-ever.

Cuvette Filling

The cuvette can be characterized as a complex system of multiple constituent elements ensuring the necessary safety level on the ISS. The first protective barrier includes the inner vessel (fig. S1.1), pistons (fig. S1.2), and the inner tube (fig. S1.3). The second and third protective barriers consist of components reported in fig. S1.4–11.

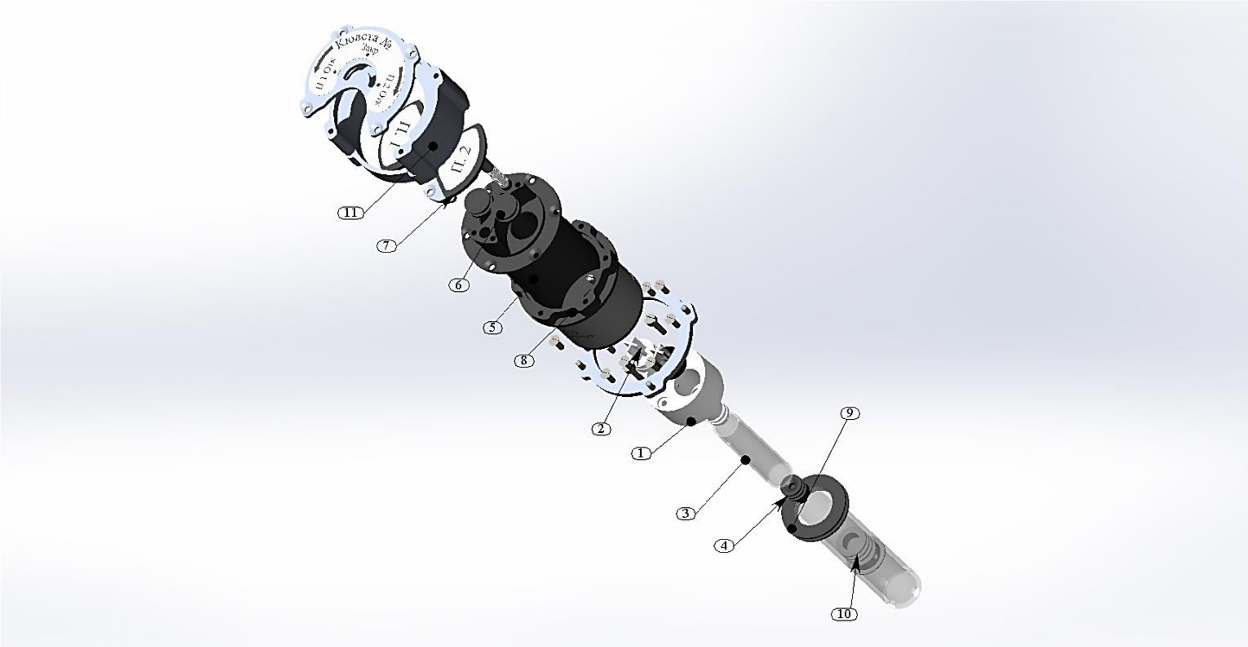

Figure S1. The cuvette used in the experiment. 1 – inner vessel; 2 – pistons; 3 – inner tube; 4 – compensator; 5 – outer case; 6 – protective pistons; 7 – pistons; 8 – protective frame; 9 – external tube; 10 – compensator; 11 – retainer. Photo credit: Stanislav V. Petrov, Laboratory for Biotechnological Research “3D Bioprinting Solutions”, Moscow, Russia

It is essential to make sure that the cuvette is completely disassembled and its parts are clean and sterile before starting to fill the cuvette with biomaterials. The cuvettes should be sterilized with 70% ethanol before each use.

Cell culture medium, fixing solution, and biomaterials incorporated in thermoreversible hydrogel should be placed into the reservoirs # 1, 2, and 3, correspondingly (fig. S2.1–3). Once the reservoir #1 (fig. S2.1) is filled with a cell culture medium, the piston (fig. S2.4) should be installed, so that the volume of the medium is 1±0.1 ml. While excessive pressure occurs in reservoir #1, a portion of the cell culture medium passes into reservoir 3 through a valve. The absence of air bubbles in the reservoir #1 should be closely monitored during the whole

procedure. After installing the piston for the cell culture medium, reservoir #3 should be washed with distilled water.

Subsequently, the piston for the fixing solution (fig. S2.5) should be installed, so that the volume of the fixing solution in reservoir 2 (fig. S2.6) is 0.5±0.1 ml. It is also necessary to avoid air bubbles in the reservoir 2. A screw with a gasket should be used for sealing the hole in the reservoir 2 (fig. S2.7). When the thermoreversible hydrogel is pre-cooled to a temperature of +8°C, 0.8±0.2 ml biomaterials-hydrogel suspension should be placed into reservoir 3 (fig. S2.8). A compensator with an opening should be installed at a distance of 4-8 mm from its end position (fig. S2.9).

Once all the filling procedures are complete, the inner vessel is placed in a centrifuge at +4℃, 2500 rpm for 5 minutes, so that the pistons for cell culture medium and fixing solution are vertically below. After the centrifugation process, the hydrogel fills all the empty spaces of the inner vessel, the compensator is adjusted to its end position, and the air bubbles together with the excessive amount of suspension exit through the compensator opening. A screw with a gasket should be used for sealing the opening (fig. S2.10). The inner vessel should be washed with distilled water after centrifugation.

The process of cuvette filling is now complete.

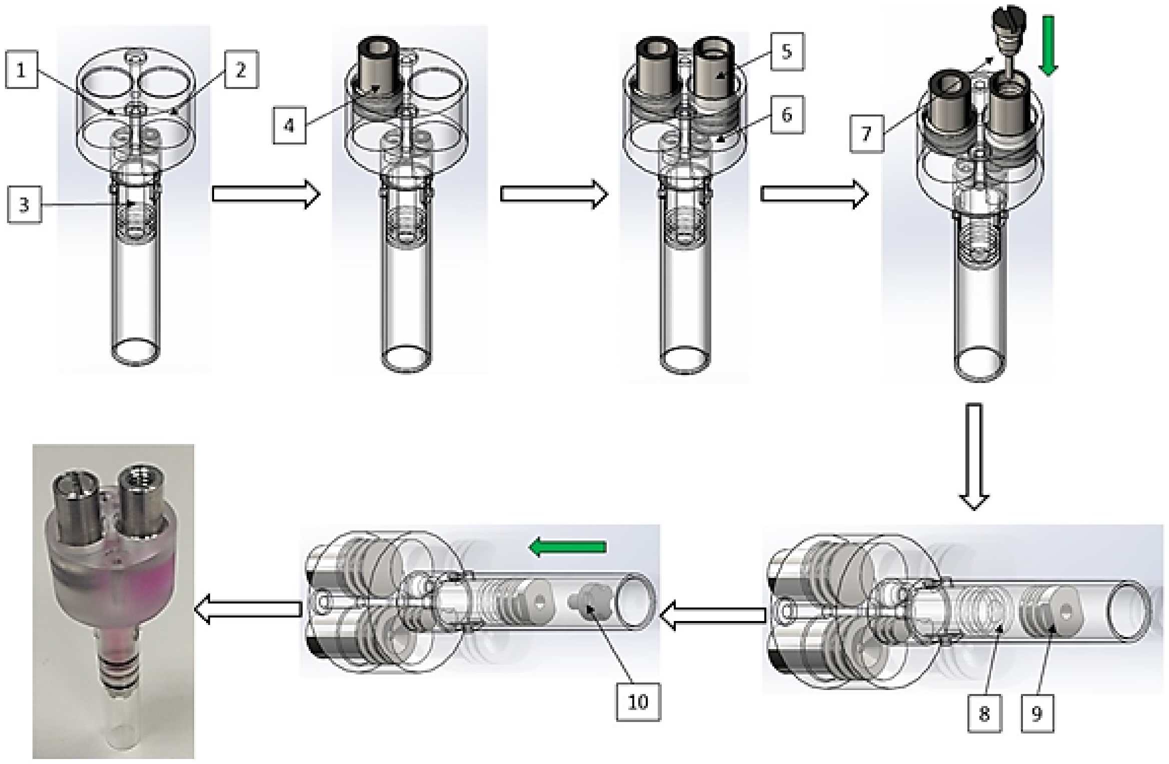

Figure S2. The step-by-step procedure of the inner vessel filling. Photo credit: Stanislav V. Petrov, Laboratory for Biotechnological Research "3D Bioprinting Solutions", Moscow, Russia

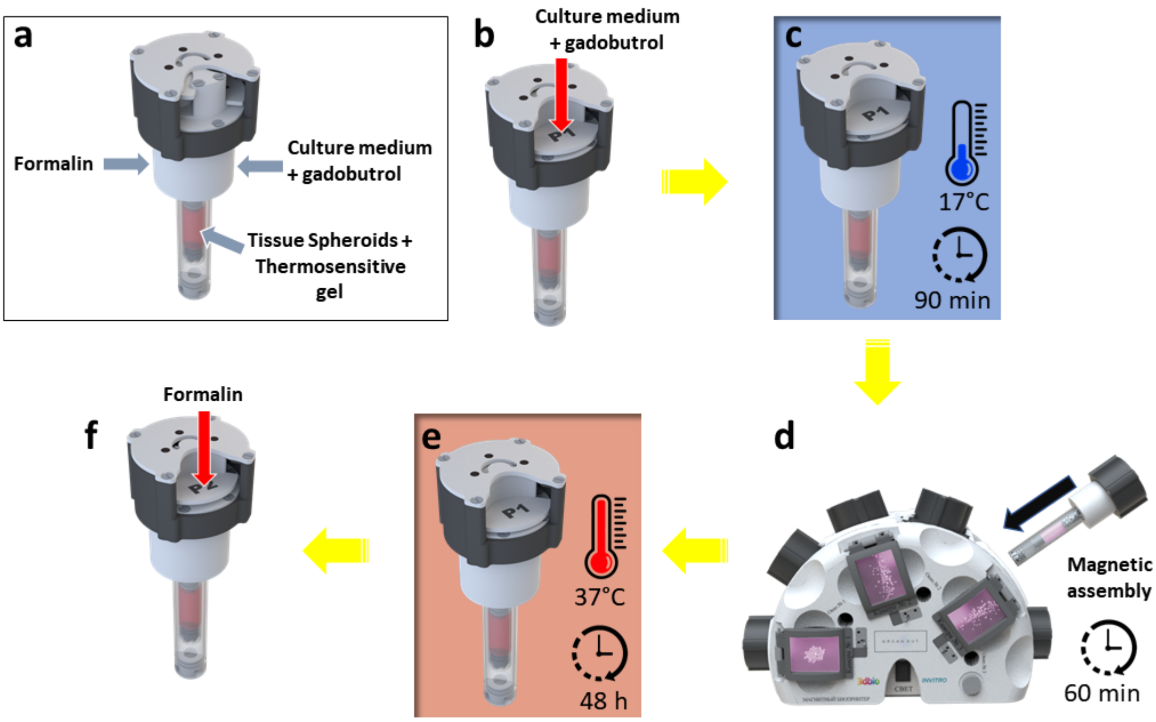

Figure S3. The detailed experimental design. a filling of cuvettes on Earth; b - the start of the space experiment after the astronaut had pressed button No. 1 to ensure injection of the medium with gadobutrol to the spheroids; c – cooling of cuvettes (90 minutes) for dissolving the thermosensitive hydrogel; d - installation of a cuvette in a magnetic bioassembler (60 minutes) for video fixation of the assembly process; e - placing the bioassembler with six cuvettes in a thermostat with a temperature of 37°C for 48 hours to provide the fusion of spheroids inside the construct; f - removing the cuvette from the magnetic bioassembler and pressing the button No. 2 to inject formalin for the fixation of the resulting construct. Photo credit: Vladislav A. Parfenov , Stanislav V. Petrov and Frederico DAS Pereira, Laboratory for Biotechnological Research “3D Bioprinting Solutions”, Moscow, Russia.

Histotypic characterization of human chondrocytes and chondrospheres.

After 14 days of cell culture under spheroid-forming conditions, the cells remain significant signs of chondrogenic differentiation and produced extracellular matrix, such as glycosaminoglycans (fig. S4c) and cartilage-specific type II collagen (fig. S4d). The cartilage-specific collagen was found to be distributed unevenly across each slice. It could result from partial loss of mature chondrocyte phenotype during the preliminary 2D-culture step that, in our case, was unavoidable since it was necessary for cell expansion.

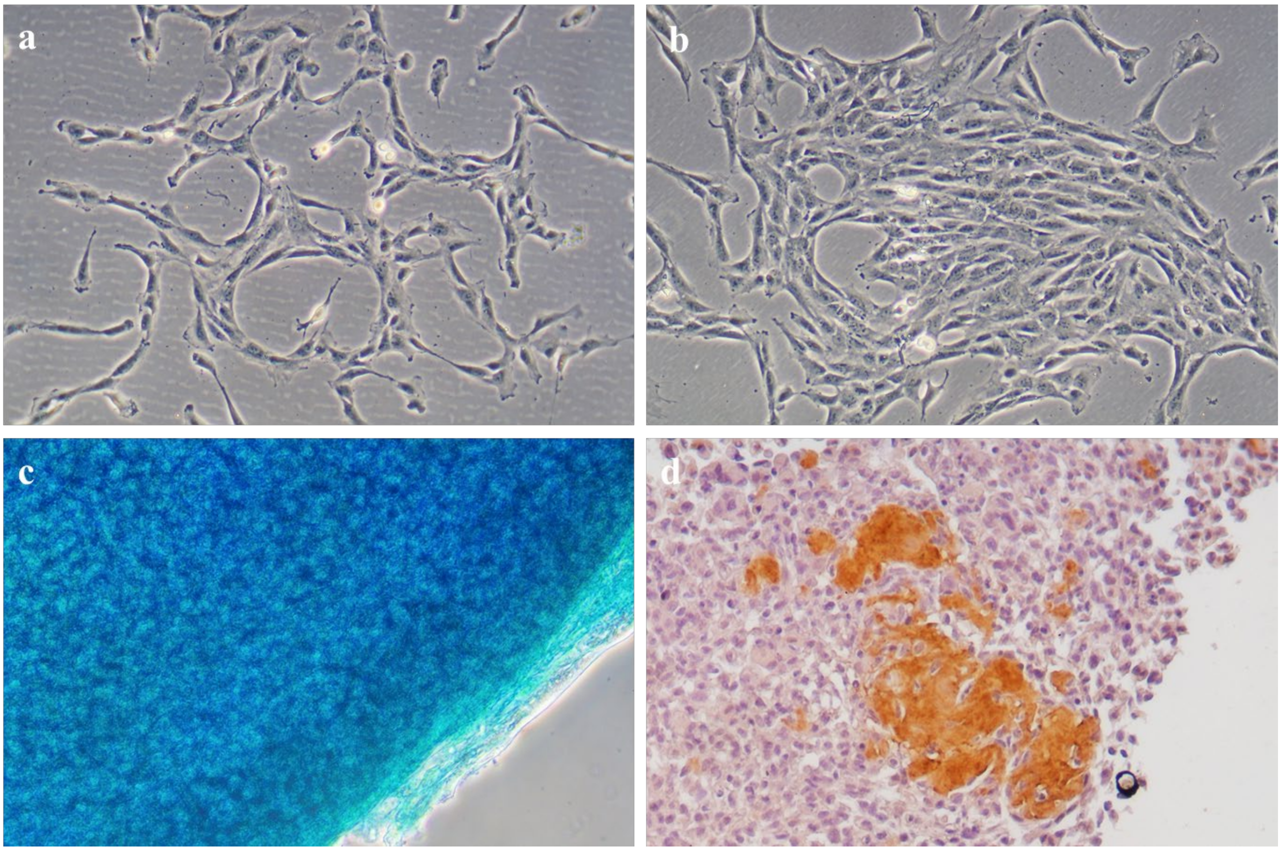

Figure S4. Histotypic characterization of human chondrocytes and chondrospheres. a-b - phase-contrast images of human chondrocytes in the 2D culture at different confluence levels. The cells appeared polygonal in shape and formed confluent monolayers after prolonged cultivation; c - alcian blue staining of chondrospheres visualized using light microscopy. Accumulation of cell-produced glycosaminoglycans in the ECM was visible; d - type II collagen expression on chondrospheres, as shown with IHC staining. Photo credit: Igor V. Vakhrushev, Laboratory for Biotechnological Research “3D Bioprinting Solutions”, Moscow, Russia

Mathematical description of assembly dynamics

The main idea of the magnetic levitation approach is to create a zone in which particles will collect under the action of magnetic forces and form ajoint construct. The experimental setup was created consisting of two permanently connected ring magnets of width L connected by the same poles. For the magnetic force to act on the particles to the center, a particular structure of the magnetic field is necessary. Since the direction of the magnetic force depends on the gradient of the square of the field, a minimum of all three spatial magnetic field strength components H_x, H_y and H_z should be observed in the center of the setup in all three directions. In this case, this condition is fulfilled: the projections of the magnetic field $\boldsymbol{H}$ on the corresponding axis are shown in fig. S5a. Under the symmetry of the structure, the remaining projections onto the central axes are zero. The magnetic field strength is determined only by the parameters of the magnets.

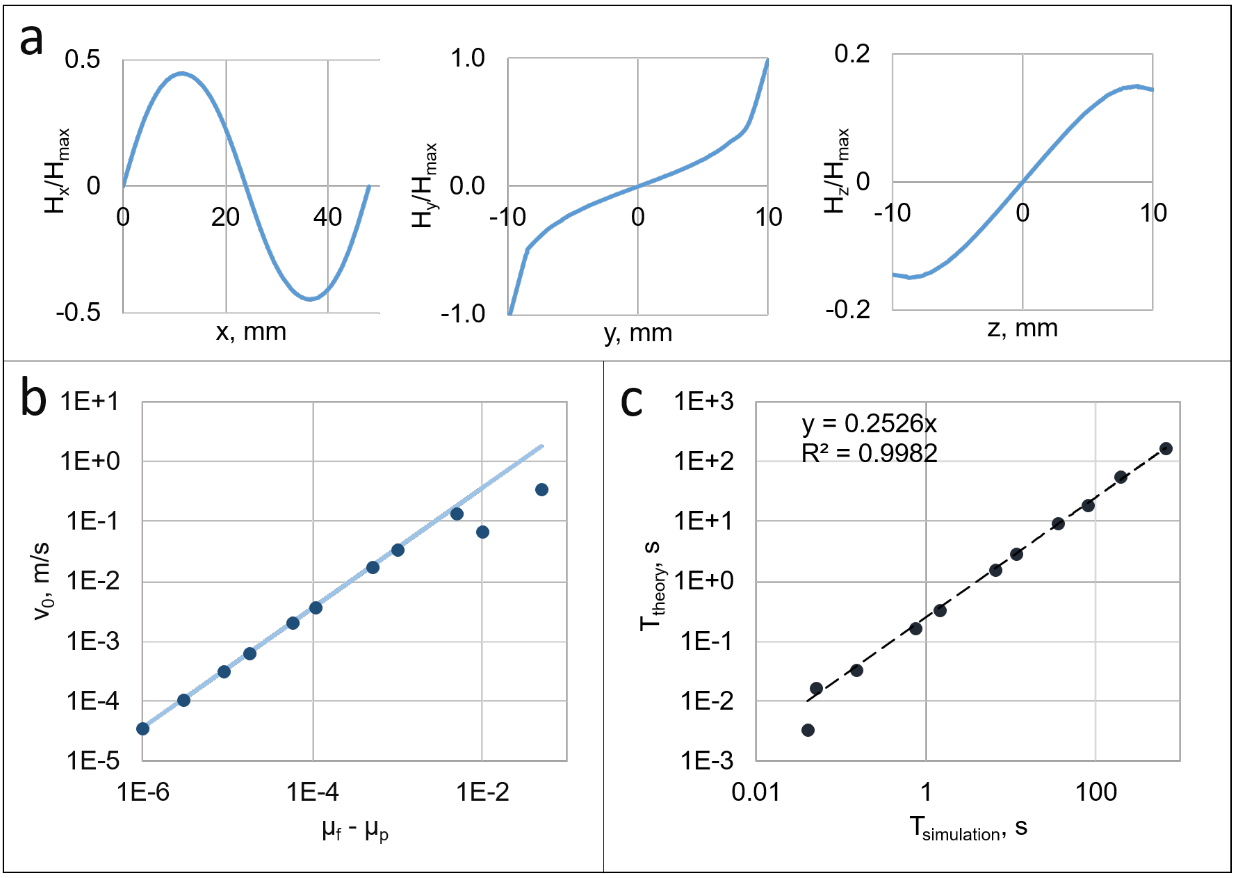

Figure S5. Parameters of the magnetic assembly. a - the projections of the magnetic field H on the corresponding axis; b - comparison of the calculated (dots) and theoretical (line) initial velocity depending on the difference in the magnetic permeabilities of the liquid and particles; c - comparison of the build time of the construct, obtained from numerical simulation, with the theoretically calculated T_ass. value for the different magnetic susceptibility of the medium

Initially, the particles in the cell are arranged randomly. It is required to understand which parameters affect the rate of convergence of the particles and determine the order of time over which the construct will be assembled.

Viscous friction acts on particles moving in a fluid. Due to the slow speed of motion and the corresponding Reynolds numbers, the viscous resistance of the medium is described by the Stokes law:

$$\boldsymbol{F}_{st} = 6\pi r\eta\boldsymbol{v},$$

where r is the radius of the particle, $\boldsymbol{v}$ is its velocity, η is the dynamic viscosity of the fluid.

The magnetic force also acts on the particles by the difference in their magnetic permeabilities and the liquid. The ratio of permeabilities determines if the force pushes the particles out of the strong magnetic field region towards the weak field or acts in the opposite direction. The effective magnetic force acting on a particle in a non-uniform magnetic field could be described as follows:

$$\boldsymbol{F}_m = 2\pi r^3\mu_0\mu_f K\nabla(\boldsymbol{H}^2),$$

where μ_0 is the magnetic constant, μ_f is the relative magnetic permeability of the liquid, μ_p is the relative magnetic permeability of the particles, and $K = \frac{\mu_p-\mu_f}{\mu_p+2\mu_f}$.

An equation for the motion of a particle in such a field provides the estimation of its dynamics. Also, we consider the movement of a particle along the x-axis in the middle of the magnets. Due to the symmetry of the structure, only the x component of the magnetic force $F_{mx} = 2\pi r^3\mu_0\mu_f K\frac{\partial(H_x^2)}{\partial x}$, and the Stokes force $F_{st} = 6\pi r\eta\frac{\partial x}{\partial t}$ will act on the particle. Since the distribution of the x component of the magnetic field H_x from the coordinate is well described by the harmonic function, the magnetic force can be represented as follows: $F_{mx} = f_m\sin\left(\frac{2\pi x}{L}\right)$, где $f_m = 2\pi r^3\mu_0\mu_f K H_{max}^2$. We write the equation of balance of forces:

$$(m + m_{ad})\ddot{x} + 6\pi r\eta\dot{x} + f_m\sin\left(\frac{2\pi x}{L}\right) = 0,$$

where m_{ad} – the additional mass entrained by the spherical particle in the liquid. Since the motion of particles is rather slow, the term $(m + m_{ad})\ddot{x}$ can be neglected compared to the other members. Then the equation reduces to the form:

$$6\pi r\eta\dot{x} = -f_m\sin\left(\frac{2\pi x}{L}\right).$$

Separating the variables and integrating, we obtain the following dependence of x-coordinate on time:

$$x(t) = \frac{L}{2\pi}\arccos\left(\frac{1 - Ee^{-\delta t}}{1 + Ee^{-\delta t}}\right), E = const,\ \delta = \frac{2f_m}{3\eta r L}.$$

The resulting equation describes the motion of a particle in a given field. The introduced value of δ is essentially a ratio of magnetic forces and viscosity forces and determines the speed of particle assembly. The constant E, which arises during integration, is determined by the initial conditions of the particle:

$$x|_{t=0} \equiv x_0 \to E = \frac{1 - \cos\left(\frac{2\pi x_0}{L}\right)}{1 + \cos\left(\frac{2\pi x_0}{L}\right)}.$$

The particle velocity can be determined by differentiating the equation of motion:

$$v(t) = -\frac{L\delta\sqrt{E}\,e^{-\delta t}}{1 + Ee^{-\delta t}}.$$

As can be seen from the relation, the initial velocity of the particle turns out to be non-zero: even in the initial state of rest under the action of a magnetic field, the particle almost instantly gains speed

$$v_0 = \dot{x}|_{\,t=0} = -\frac{L\delta}{4\pi}\sin\left(\frac{2\pi x_0}{L}\right).$$

A computer simulation of the dynamics of particle assembly in a viscous fluid under the action of a magnetic field was carried out. The differences in the magnetic susceptibilities of the particles and the liquid varied in our simulation, while the other parameters of the system remained constant. As the initial position of the particles, $x_0 = L/4$ was chosen, which corresponds to $E = 1$. In this case, the expression for the dependence of the coordinate on time is simplified:

$$x(t) = \frac{L}{2\pi}\arccos\left(\tanh\left(\frac{\delta t}{2}\right)\right),$$

$$v(t) = -\frac{L\delta e^{-\delta t}}{1 + e^{-\delta t}}.$$

Since the concentration of a paramagnet salt in a liquid varied, the time for particle central gathering in the magnetic installation depended on its magnitude. One of the characteristic values for given initial conditions is the initial velocity of motion $v_0 = -\frac{L\delta}{4\pi}$. Figure S4b shows a comparison of the calculated (dots) and theoretical (line) initial velocity depending on the difference in the magnetic permeabilities of the liquid and particles. In general, there is a good agreement between theory and simulation, and the difference in speeds and with the most significant difference in magnetic permeabilities is explained by the fact that the motion of particles is fast and does not go beyond the accepted approximation.

As can be seen from the formula, v_0 is directly proportional to the value of δ, which is the main criterion for build time. Then the build time order can be defined as

$$T_{ass}{\sim}\pi/\delta.$$

Figure S4c shows a comparison of the build time of the construct, obtained from numerical simulation, with the theoretically calculated T_ass value for the different magnetic susceptibility of the medium. As can be seen from the graph, the ratio between the calculated and theoretical values remains constant – the linear trend line fits very well. Thus, knowing the exact assembly time for a specific magnetic susceptibility of the medium, one can accurately estimate the assembly time of the same system with a different magnetic susceptibility.

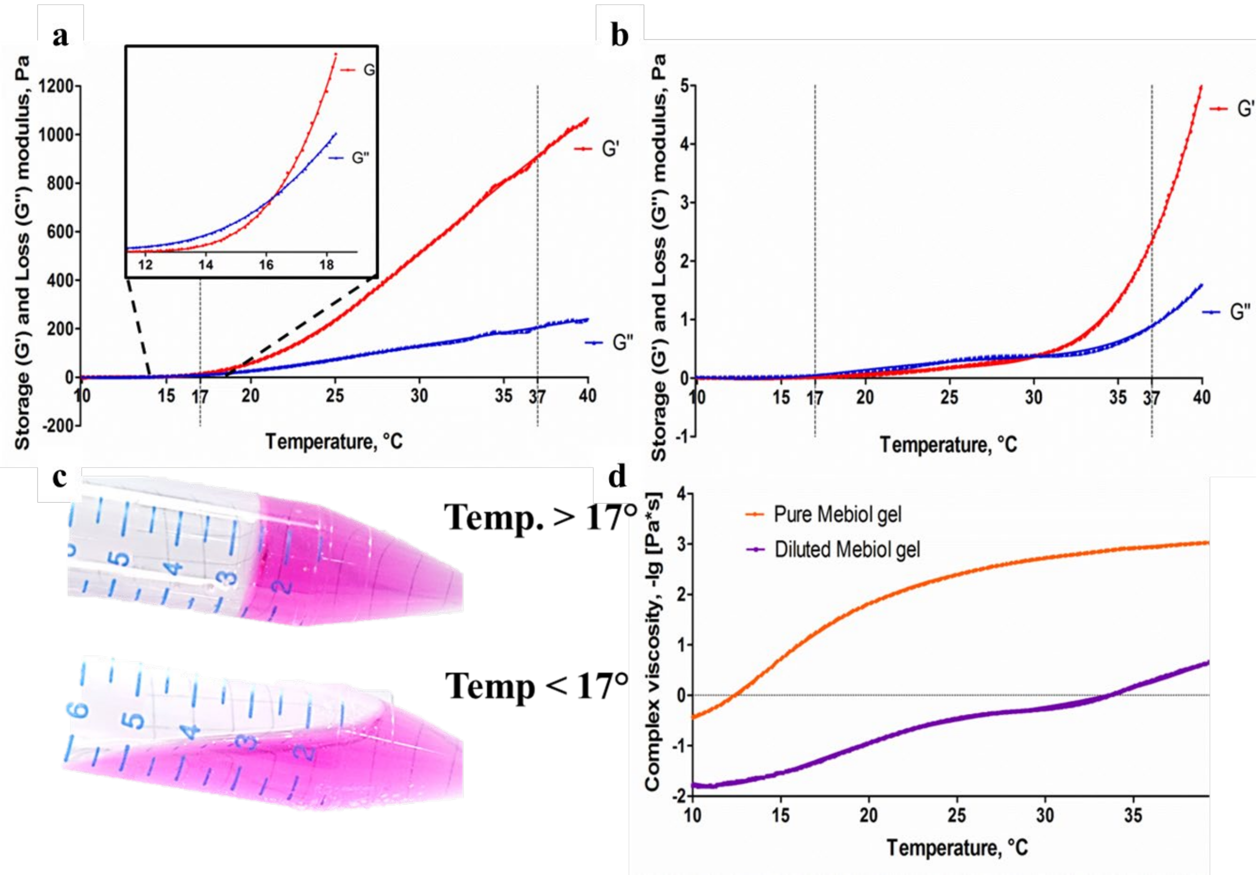

Figure S6. Rheological parameters of thermoreversible hydrogel used for chondrospheres delivery to the ISS. a - characteristics of pure NIIPAM-PEG thermoreversible hydrogel; b - characteristics of the diluted thermosensitive hydrogel in the compound of biofabrication medium; c – phase transition of NIIPAM-PEG thermoreversible hydrogel; d - viscosity of pure and diluted hydrogel. Photo credit: Frederico DAS Pereira, Laboratory for Biotechnological Research “3D Bioprinting Solutions”, Moscow, Russia

Effects of Mebiol gel on biomechanical properties of tissue spheroids

The main benefit of using a micro-scale parallel-plate compression testing system is the possibility of measuring biomechanical properties of the whole tissue spheroid without reference to the fixed point of the sample. This approach allows quantifying the overall biomechanical profile of every single tissue spheroid as accurately as possible. We did not observe any significant difference between the biomechanics of tissue spheroids incubated both in Mebiol gel and culture media. However, tissue spheroids after incubation in Mebiol gel demonstrated slightly increased elastic modulus compared to the control group: 3.51±0.80 kPa and 3.38±0.79 kPa, correspondingly (fig. S7).

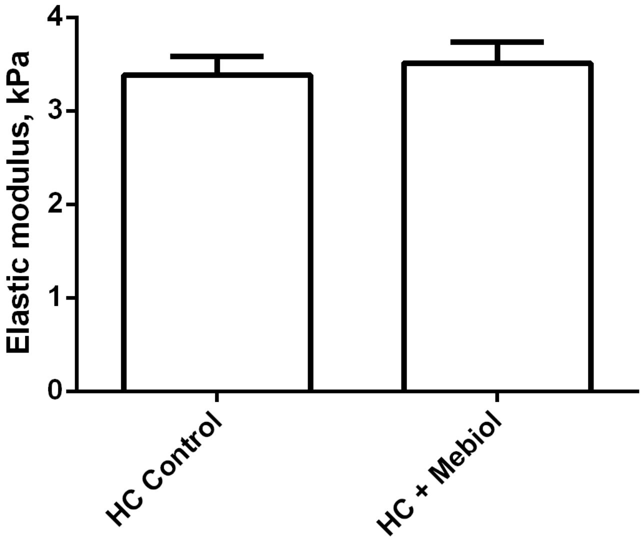

Figure S7. The elasticity of tissue spheroids in the presence or w/o thermoreversible hydrogel.

Effect of gadobutrol exposure on cell viability

The effect of gadobutrol exposure on the viability of human chondrocytes was examined by the resazurin cell viability assay. The concentrations of gadobutrol less than 12.5mM do not affect cell viability, while 50mM Gd^{3+} reduced cell viability by 84% at 24 hours (fig. S8).

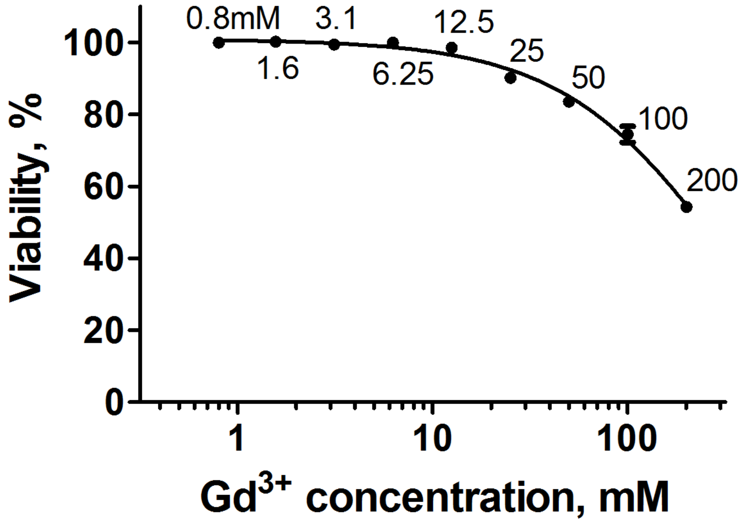

Figure S8. The viability of human chondrocytes after exposure to different concentrations of gadobutrol for 24 hours.

Video S1. Construct assembly kinetics simulation (front view)

Video S2. Construct assembly kinetics simulation (side view)

Video S3. Construct assembly kinetics simulation (top view)

Video S4. Construct assembly kinetics simulation (isometry)

Video S5. Construct assembly from chondrospheres in space experiment

Video S6. Assembly of cuvette
